# Pattern of cause-specific childhood mortality in a malaria endemic area of Burkina Faso

**DOI:** 10.1186/1475-2875-5-47

**Published:** 2006-06-08

**Authors:** Gaël P Hammer, Florent Somé, Olaf Müller, Gisela Kynast-Wolf, Bocar Kouyaté, Heiko Becher

**Affiliations:** 1 Department of Epidemiology, Institute for Tropical Hygiene and Public Health, University of Heidelberg, Germany; 2 Centre de Recherche en Santé de Nouna (CRSN), Nouna, Burkina Faso; 3 Institute for Medical Biostatistics, Epidemiology and Informatics, University of Mainz, Germany

## Abstract

**Background:**

Reliable mortality data are a prerequisite for planning health interventions, yet such data are often not available in developing countries, particularly in sub-Saharan Africa (SSA). Demographic surveillance systems (DSS) implementing the verbal autopsy (VA) method are the only possibility to observe cause-specific mortality of a population on a longitudinal basis in many countries.

**Methods:**

This paper reports all-cause and cause-specific mortality rates in children under the age of five years from 1999 until 2003 in a malaria holoendemic area of north-western Burkina Faso. The DSS of the Nouna Health Research Centre, in which VA data were analysed, covers a rural population of about 30,000 (41 villages) and an urban population of about 25,000 (Nouna town).

**Results:**

A total of 1,544 deaths were analysed, 87 (6%), 225 (14%), 317 (21%) and 915 (59%) of which occurred in the periods < 1 month, 1–5 months, 6–11 months and 1–4 years respectively. All cause mortality rates of children under five years were higher in the rural than the urban area (34 vs 24 per 1,000 person-years) and in the rainy than the dry season (35 vs 29 per 1,000 person-years). Malaria was the most frequent diagnosis (42%) with peak mortality rates in infants aged 6–11 months.

**Conclusion:**

Malaria is the most important cause of death in this remote area of SSA, even considering the low specificity of malaria diagnosis in young children. Strengthening the existing malaria control tools is of prime importance to reduce the high childhood mortality in the endemic areas of SSA.

## Background

Infant and childhood mortality remains high in the poor populations of the developing world [1,2]. About 10.6 million children under the age of five years are dying each year, with the great majority of deaths being preventable by low-cost public health interventions [2-4]. Roughly 40 percent of childhood deaths are estimated to occur in the neonatal period (the first 4 weeks of life), with this proportion being considerably lower for regions with high absolute rates such as sub-Saharan Africa (SSA) [5]. On a global scale, only six causes account for three-quarters of childhood deaths: pneumonia (19%), diarrhoea (18%), malaria (8%), neonatal pneumonia or sepsis (10%), preterm delivery (10%) and asphyxia at birth (8%) [3]. Malnutrition is a risk factor suspected to be an additional underlying cause in about half of all cases of childhood deaths [6].

The Millennium Development Goal for child survival will not be met without seriously addressing childhood mortality through known and effective interventions [4,7]. However, the efficacy of child survival programmes depends on accurate information on all-cause as well as cause-specific mortality, which is often lacking from developing countries [8]. In most countries of SSA, there are no vital registration systems and most childhood deaths occur at home [1,9]. In such areas, demographic surveillance systems (DSS) are often the only reliable source for all-cause and cause-specific mortality pattern in childhood [10].

DSS sites, also called population monitoring laboratories, exist in a number of SSA countries [10]. Besides providing reliable information on trends in all-cause mortality, most DSS sites use verbal autopsy methods to ascribe causes of death [10-12]. This method works well for diseases with clearly defined symptoms, such as measles and tetanus, but less so for diseases with overlapping symptoms such as malaria and pneumonia [13,14], particularly in children. Despite these limitations, verbal autopsies are the only way to study the causes of deaths in rural SSA and this method has consequently been applied in a number of countries [15-23].

This publication reports cause-specific childhood mortality rates from a DSS site in rural north-western Burkina Faso, an area of intense malaria transmission.

## Study population and methods

### Study area

The research zone of the *Centre de Recherche en Santé de Nouna *(CRSN) in Nouna Health District, north-western Burkina Faso, covers a rural area (41 villages, 30,000 people) and an urban area (Nouna town, 25,000 inhabitants).

The Nouna area is a dry orchard savannah, populated mainly by subsistence farmers of different ethnic groups. Childhood mortality is high in the area, with substantial variation between villages [24]. The frequent occurrence of malnutrition in young children is a major determinant of the high childhood mortality in the area [6,25].

Malaria is holoendemic but highly seasonal in the rural CRSN study area where the annual Entomological Inoculation Rate (EIR) varies between 100 and 1000 between the different study villages [26,27]. In Nouna town, the capital city of Nouna Health District, malaria is showing a mesoendemic transmission pattern [28]. Formal health services are limited to a few rural health centres and the district hospital in Nouna town. Chloroquine is still the official first-line treatment for uncomplicated malaria in Burkina Faso. The clinical failure rate at day 14 after chloroquine treatment has been shown to be 10% in the rural CRSN study area in 2001 and 53% in the urban CRSN study area by 2003 [29,30]. There was a rapid increase in chloroquine resistance observed in the rural area since 2001 (unpublished data).

### Nouna DSS study population and cause of death assessment

The Nouna DSS started operating in 1992. The town of Nouna is included in the DSS since mid-2000. Since 1998, specialized field staff systematically visits every household in a three months cycle, and records vital and demographic events: births, deaths, and in and out migration [24,31,32]. The collection of causes of death by verbal autopsy (VA) started in 1993, but due to the former passive vital event registration system data were incomplete until 1998. This publication reports the cause-specific mortality of the years 1999 to 2003 of all individuals born between January 1^st^, 1994, and December 31^st^, 2003, and registered in the DSS. The Nouna DSS encompasses the population of 39 villages (January 1999 until December 2003) and, since July 2000, the town of Nouna and two more villages.

Verbal autopsy data are collected by trained field staff who systematically visit the households of deceased persons in the CRSN study area. A standardized questionnaire, developed at CRSN, is usually applied between one and six months after the death and after having obtained oral informed consent. The questionnaire covers demographic data and the clinical history before the death occurred (e.g. accidents, manifestation and duration of specific symptoms, treatment seeking behaviour and treatment). Some 5–10% of these questionnaires are systematically checked by the supervisors of field staff, which also includes field visits and repeat interviews. These questionnaires are then read by two experienced local physicians out of a pool of locally available and specifically trained physicians who assign a definite cause of death. In case of discordance, a third physician is getting involved. At least two physicians have to agree on the final diagnosis.

### Statistical analysis

Data are categorized by gender, age group (< 1 month, 1–5 months, 6–11 months, 12–59 months, all infants and all children), calendar year (1999–2003), setting (urban, rural) and season (rainy, dry).

The dry season is defined here as the period from November 1^st ^until May 31^st^. This definition was chosen mainly to be consistent with previous publications, although the transmission of diseases, especially of malaria, lags behind the period of rainfall.

First, the completeness of VA data was investigated by tabulating the percentage of missing values by categories of each variable. Additionally, joint effects of these variables on the probability of missing VA questionnaires were analysed by multivariate logistic regression.

Secondly, overall and cause-specific mortality rates were computed by age, season and setting. Causes of death were grouped into the following categories: malaria, acute respiratory infections, acute gastrointestinal infections, other infectious diseases, neonatal causes, non-natural causes, other non-infectious diseases, other causes, ill-defined causes, and "missing cause of death". Mortality rate ratios (RR) and corresponding exact confidence intervals were also calculated [33].

Finally, multivariate Poisson regression was used to model the joint effect of gender, season, setting and age group on all-cause and malaria childhood mortality.

All analyses were performed using SAS 9.1 software [34].

## Results

A total of 25,406 children born between January 1994 and December 2003 were registered in the DSS. 611 (2.4 %) were excluded from the analyses because of missing date of entry into the DSS, 1054 (4.1%) because they entered the DSS area after their 5^th ^birthday, 637 (2.5%) because they left the DSS before 1999, 26 (0.1%) because they entered the DSS after 2003, 95 (0.4%) because of inconsistent date values, and 4 because of missing information on season of death, leaving 22,979 in the analysis cohort. The mid-year population in 2003 was 11,155 children under five years of age. The mean observation time of the children was 2.11 years, and total observation time was 48.495 person-years. 1,544 children died during the observation period.

Table 1 presents the number of deaths, and the proportion of collected VA questionnaires by age group, year, age class, setting, and season. A verbal autopsy questionnaire has been completed for 1.208 (78.2%) of all deceased. The probability of VA being collected does not depend on gender or setting, but on age, with less VA diagnoses available from deaths which occurred during the neonatal period. There is, furthermore, a trend towards more completed VA diagnoses in the more recent years. Finally, less VA diagnoses are missing for children deceased in the rainy season than in the dry season.

**Table 1:** Number of deaths in children under five and existence of corresponding verbal autopsy (VA) information by sex, year, age group, setting and season in the period 01.01.1999 to 31.12.2003 in the DSS of Nouna, Burkina Faso

		Total (N)	Total person-years	VA collected (%)	p^1^
Gender	Female	738	24,712.1	78.46	0.99
	Male	806	24,695.5	78.04	

Date of death	1999	226	6,245.2	74.34	0.04
	2000	339	10,567.8	74.34	
	2001	340	10,927.1	80.59	
	2002	279	10,895.9	81.72	
	2003	360	10,771.6	79.44	

Age	Neonates (< 1 month)	87	929.8	68.97	< 0.01
	Infants (1–5 months)	225	4,672.3	75.56	
	Infants (6–11 months)	317	5,392.3	78.86	
	Children (1–4)	915	38,413.3	79.56	

Setting	Villages	1,237	36,735.6	77.85	0.87
	Nouna town	307	12,672.1	79.80	

Season	rainy (Jun-Oct)	713	20,484.9	80.79	0.02
	dry (Nov-May)	831	28,922.8	76.05	

Total		1,544	49,407.7	78.23	

^1 ^overall variable effect

Of all 1,544 deaths, 87 (5.6%) occurred in the neonatal period, 225 (14.6%) during the first half of infancy, 317 (20.5%) during the second half of infancy, and 915 (59.3%) in the 2^nd ^to 5^th ^year of life.

Death rates in neonates, infants, and children under five are 93.57, 57.21 and 31.25 per 1000 person-years, respectively. Table 2 shows all-cause and cause-specific mortality rates per 1,000 person-years by sub-area (rural and urban), season (dry and rainy) and age group. Table 3 summarizes the strong variation of all-cause and malaria mortality by area and season in a univariate fashion.

**Table 2:** Cause-specific mortality rates per 1000 person-years in children under five 1999–2003 in the DSS of Nouna, Burkina Faso

	Rural Area						Urban Area					
	
	Neonates (< 1 m)	Infants (1–5 m)	Infants (6–11 m)	All infants	Children (1–4 yrs)	Under five	Neonates (< 1 m)	Infants (1–5 m)	Infants (6–11 m)	All infants	Children (1–4 yrs)	Under five
Rainy Season												

All deaths	28	82	137	247	304	551	6	12	41	59	103	162
All causes	88.33 (58.7–123.3)	57.17 (45.5–70.1)	78.45 (65.9–92.1)	70.62 (62.1–79.7)	25.83 (23.–28.8)	36.09 (33.1–39.2)	63.10 (23.0–117.8)	27.43 (14.2–44.2)	69.85 (50.1–92.5)	52.71 (40.1–66.9)	25.14 (20.5–30.2)	31.05 (26.5–36.0)
Malaria	9.46 (1.9–20.7)	27.19 (19.3–36.2)	48.10 (38.4–58.9)	36.03 (30.3–42.6)	12.91 (10.9–15.0)	18.21 (16.1–20.4)	21.03 (2.4–50.6)	11.43 (3.7–22.2)	37.48 (23.5–54.3)	25.91 (17.3–36.0)	15.62 (12.0–19.6)	17.83 (14.4–21.6)
ARI	3.15 (0.0–8.8)	5.58 (2.4–9.8)	3.44 (1.3–6.4)	4.29 (2.4–6.6)	1.02 (0.5–1.6)	1.77 (1.2–2.5)	10.52 (0.1–29.3)	.	1.70 (0.0–4.7)	1.79 (0.2–4.3)	0.24 (0.0–0.7)	0.58 (0.1–1.3)
AGI	3.15 (0.0–8.8)	2.79 (0.7–5.7)	4.58 (2.0–8.0)	3.72 (2.0–5.9)	3.14 (2.2–4.2)	3.27 (2.4–4.2)	.		6.82 (1.8–14.0)	3.57 (1.0–7.3)	2.20 (1.0–3.8)	2.49 (1.3–4.0)
Neonatal cause	41.01 (21.8–65.1)	.	.	5.43 (3.3–8.1)	.	0.85 (0.5–1.4)	10.52 (0.1–29.3)	.	.	0.89 (0.0–2.5)		0.19 (0.0–0.5)
Non natural	.	.	.	.	0.59 (0.2–1.1)	0.46 (0.2–0.8)	.		.	.	0.73 (0.1–1.6)	0.58 (0.1–1.3)
Other causes	.	8.37 (4.30 – 13.50)	2.86 (0.90 – 5.60)	4.86 (2.80 – 7.40)	2.55 (1.70 – 3.50)	3.08 (2.30 – 4.00)	10.52 (0.10 – 29.30)	2.29 (0.00 – 6.40)	6.82 (1.80 – 14.00)	5.36 (2.00 – 10.00)	1.71 (0.70 – 3.10)	2.49 (1.30 – 4.00)
Ill-defined	6.31 (0.7–15.2)	2.09 (0.4–4.6)	3.44 (1.3–6.4)	3.15 (1.6–5.2)	0.93 (0.5–1.5)	1.44 (0.9–2.1)	.	4.57 (0.5–11.0)	6.82 (1.8–14.0)	5.36 (2.5–11.3)	.	1.15 (0.4–2.1)
Missing	25.24 (10.9–44.2)	11.15 (6.4–17.1)	16.03 (10.6–22.4)	14.87 (11.1–19.1)	4.67 (3.5–6.0)	7.01 (5.7–8.4)	10.52 (0.1–29.3)	9.14 (2.5–18.7)	10.22 (3.7–19.1)	9.83 (4.9–16.1)	4.64 (2.8–6.9)	5.75 (3.9–7.9)

Dry Season												

All deaths	51	109	103	263	423	686	2	22	36	60	85	145
All causes	128.11 (95.4–165.2)	51.45 (42.2–61.5)	44.25 (36.1–53.2)	54.29 (47.9–61.0)	25.44 (23.1–27.9)	31.95 (29.6–34.4)	16.69 (1.9–40.2)	32.24 (20.2–46.7)	49.21 (34.5–66.3)	39.12 (29.8–49.5)	14.36 (11.5–17.5)	19.45 (16.4–22.7)
Malaria	2.51 (0.0–7.0)	15.10 (10.3–20.7)	20.62 (15.2–26.8)	16.72 (13.3–20.5)	8.96 (7.6–10.5)	10.71 (9.4–12.1)	.	14.65 (7.0–24.5)	20.50 (11.5–31.7)	16.30 (10.5–23.1)	6.59 (4.7–8.8)	8.58 (6.6–10.8)
ARI	7.54 (1.5–16.5)	7.55 (4.3–11.5)	2.58 (0.9–4.8)	5.16 (3.3–7.3)	1.74 (1.2–2.4)	2.52 (1.9–3.2)	.	4.40 (0.9–9.6)	2.73 (0.3–6.6)	3.26 (1.0–6.3)	1.69 (0.8–2.8)	2.01 (1.1–3.1)
AGI	2.51 (0.0–7.0)	2.83 (1.0–5.3)	4.73 (2.4–7.8)	3.72 (2.2–5.6)	3.79 (2.9–4.8)	3.77 (3.0–4.6)	.	1.47 (0.0–4.1)	8.20 (3.0–15.3)	4.56 (1.8–8.2)	1.18 (0.5–2.1)	1.88 (1.0–2.9)
Neonatal cause	47.73 (28.7–70.8)	.	.	3.92 (2.4–5.8)	.	0.89 (0.5–1.3)	8.34 (0.1–23.2)		.	0.65 (0.1–3.1)		0.13 (0.0–0.4)
Non natural	2.51 (0.0–7.0)	.	0.86 (0.1–2.1)	0.62 (0.1–1.4)	0.48 (0.2–0.8)	0.51 (0.3–0.8)	.		1.37 (0.0–3.8)	0.65 (0.0–1.8)	0.17 (0.0–0.5)	0.27 (0.0–0.6)
Other causes	10.05 (2.70 – 20.60)	9.91 (6.10 – 14.50)	3.01 (1.20 – 5.40)	6.61 (4.50 – 9.00)	3.49 (2.60 – 4.40)	4.19 (3.40 – 5.10)	.	2.93 (0.30 – 7.10)	2.73 (0.30 – 6.60)	2.61 (0.70 – 5.30)	1.35 (0.60 – 2.40)	1.61 (0.80 – 2.60)
Ill-defined	10.05 (2.7–20.6)	1.89 (0.5–3.9)	2.15 (0.7–4.2)	2.68 (1.4–4.3)	1.26 (0.8–1.8)	1.58 (1.1–2.2)	8.34 (0.1–23.2)	1.47 (0.0–4.1)	1.37 (0.0–3.8)	1.96 (0.4–4.3)	0.34 (0.0–0.8)	0.67 (0.2–1.3)
Missing	45.22 (26.8–67.7)	14.16 (9.5–19.6)	10.31 (6.6–14.7)	14.87 (11.6–18.5)	5.71 (4.6–6.9)	7.78 (6.6–9.0)	0.00 (0.0-0.0)	7.33 (2.4–14.3)	12.30 (5.6–21.0)	9.13 (5.0–14.3)	3.04 (1.8–4.6)	4.29 (2.9–5.9)

ARI = acute respiratory infections

AGI = acute gastrointestinal infections

m = months

**Table 3:** All-cause and malaria mortality rates per 1000 person-years and RRs (with 95% CI) for infants and under five by area in the DSS of Nouna, Burkina Faso

		Infants					Under five				
		
		Deaths	Person-years	Rate	RR	95%-CI	Deaths	Person-years	Rate	RR	95%-CI
All-cause	Overall	629	10994.34	57.21			1544	49407.66	31.25		
	
	Villages	510	8.341.03	61.14	1.00		1237	36735.55	33.67	1.00	
	Nouna	119	2.653.31	44.85	0.73	(0.60 – 0.90)	307	12672.11	24.23	0.72	(0.63 – 0.82)
	
	Rainy season	306	4.616.90	66.28	1.00		713	20484.91	34.81	1.00	
	Dry season	323	6.377.44	50.65	0.76	(0.65 – 0.90)	831	28922.75	28.73	0.83	(0.75 – 0.91)

Malaria	Overall	261	10994.34	23.74			665	49407.66	13.46		
	
	Villages	207	8.341.03	24.82	1.00		508	36735.55	13.83	1.00	
	Nouna	54	2.653.31	20.35	0.82	(0.60 – 1.11)	157	12672.11	12.39	0.90	(0.74 – 1.07)
	
	Rainy season	155	4.616.90	33.57	1.00		371	20484.91	18.11	1.00	
	Dry season	106	6.377.44	16.62	0.50	(0.38 – 0.64)	294	28922.75	10.17	0.56	(0.48 – 0.66)

All-cause infant and under five mortality are lower in the urban compared to the rural area (61 vs 45 and 34 vs. 24 per 1000 person-years, corresponding to RR = 0.73, 95% CI: 0.60–0.90 and RR = 0.72, 95% CI: 0.63–0.82). Comparing mortality by season, all-cause infant and under five mortality are lower during the dry compared to the rainy season (66 vs 51 and 35 vs 29, corresponding to RR = 0.78, 95% CI: 0.67–0.91 and RR = 0.83, 95% CI: 0.75–0.91).

Malaria is the far most frequent VA diagnosis (42.2%) with an overall rate of 12.39 per 1000 child-years in the urban and 13.83 in the rural study area. Under five mortality attributed to malaria strongly peaks in the second half of infancy. It is non-significantly lower in the urban than in the rural setting (RR = 0.82, 95% CI 0.61–1.11 in infants and RR = 0.9, 95% CI 0.74–1.07 in under fives), and significantly lower during the dry than to the rainy season (RR = 0.50, 95% CI 0.38–0.64 in infants and RR = and 0.56, 95% CI 0.48–0.66 in under fives).

Mortality due to acute respiratory infections peaks during the first half of infancy and tends to be higher in the rural compared to the urban study area as well as during the dry compared to the rainy season. In contrast, mortality attributed to acute gastrointestinal infections peaks during the second half of infancy without marked differences by season. Other causes of mortality, including other infectious diseases, neonatal causes and non-natural causes, are less often diagnosed and do not show a clear pattern with regard to setting or season.

The multivariate analysis of risk factors for overall and malaria mortality is given in Table 4. No gender differences are observed. Both all-cause and malaria mortality are significantly higher in the rainy compared to the dry season (RR = 1.19 and 1.75), lower in Nouna town than in the surrounding villages (RR = 0.75 and 0.92), and significantly higher in infants than in older children.

**Table 4:** Rate Ratios for all-cause and malaria childhood mortality 1999–2003 in the Nouna DSS, based on multivariate Poisson Regression

	Variable	Level	Rate Ratio	95% Confidence Interval	p > χ^2^
All causes	Gender	Male (vs. Female)	1.10	(1.00 – 1.22)	0.06
	Season	Dry (vs. Rainy)	0.83	(0.75 – 0.92)	< .01
	Setting	Nouna town (vs. Villages)	0.75	(0.66 – 0.86)	< .01
	Age Class	Neonates (< 1 month) (vs. Children 1–4)	3.99	(3.21 – 4.98)	< .01
		Infants (1–5 months) (vs. Children 1–4)	2.01	(1.74 – 2.32)	< .01
		Infants (6–11 months) (vs. Children 1–4)	2.45	(2.15 – 2.78)	< .01

Malaria	Gender	Male (vs. Female)	0.99	(0.85 – 1.15)	0.90
	Season	Dry (vs. Rainy)	0.57	(0.49 – 0.66)	< .01
	Setting	Nouna town (vs. Villages)	0.92	(0.77 – 1.10)	0.34
	Age Class	Neonates (< 1 month) (vs. Children 1–4)	0.88	(0.39 – 1.97)	0.75
		Infants (1–5 months) (vs. Children 1–4)	1.76	(1.39 – 2.22)	< .01
		Infants (6–11 months) (vs. Children 1–4)	2.94	(2.46 – 3.52)	< .01

## Discussion

Although some data on all-cause childhood mortality are available in most countries of sub-Saharan Africa, only few countries have DSS sites which can provide additional data on cause-specific mortality. New survey instruments for VA collection [35] have recently been proposed, which might improve the availability of data in future. A number of studies have already been published on the pattern of all-cause childhood mortality in the CRSN study area of rural Burkina Faso. Findings have highlighted the variability of mortality between villages [24], shown an age-specific seasonality of mortality pattern [31,36] and demonstrated a strong association between the high prevalence of malnutrition in young children and mortality in the this study area [25]. This is the first study to report cause-specific childhood mortality from the study area, using the verbal autopsy method, and which compares a neighbouring rural and urban area.

A verbal autopsy questionnaire could be completed for 1.208 children under the age of five years, which are 78% of all 1.544 deceased during the study period. The investigation of factors influencing this proportion revealed that there were no differences by gender or setting, and that completeness increased over the years. Due to the delay between death and VA collection, and the fact that people are more frequently not at home during the rainy season because they work on the fields, less VA could be collected for deaths occurring in the dry season. VA diagnoses of older children are collected more often than those of children who have died early, likely due to cultural reasons (people do not easily talk about death of young children). The authors have no reason to believe that there is any bias in VA data collection on DSS reported deaths for other reasons.

The decrease in mortality from birth until age 5 is consistent with the pattern of mortality in comparable high-mortality countries [1,17,19]. However, the proportion of neonatal deaths on all deaths under five years of age has been estimated to be around 24% in Africa. An estimate for SSA is not provided and is likely to be around 20% [5]. The finding of only 6% of deaths occurring during the neonatal period in the Nouna area is still much lower than that, and is likely to be explained by three reasons (i) reporting bias due to socio-cultural factors (e.g. families do not like to mention children which have died during the first month) as well as (ii) by a real lower proportion of neonatal deaths in remote rural SSA areas [5,37] and (iii) by the fact that Burkina Faso has a childhood mortality which is higher than that of SSA in total. Reporting bias is likely to be stronger in DSS sites which collect data every three months, like the one of Nouna, compared to DSS sites with ongoing vital event registration (e.g. The Gambia) [5,19,31].

All-cause childhood mortality was lower in the urban setting compared to the rural study area. This is consistent with findings from other studies in sub-Saharan Africa and is likely attributed to a number of factors: 1) better access to health services, 2) better hygiene due to a safer water supply, 3) better education and communication, 4) lower malaria transmission intensity, and 5) lower prevalence of malnutrition in urban compared to rural areas [9,16,25].

Malaria is by far the most frequent diagnosis for childhood deaths in this study which compares well to a number of other malaria endemic areas in SSA [11]. However, as a post-mortem diagnosis, malaria was much more frequent compared to ARI, the worldwide most important killer disease in young children [1]. There are three likely explanations for this finding: misclassification bias, vaccination coverage, and increasing Chloroquine resistance [29,30]. Misclassification might result from the tendency of physicians working in areas of high malaria transmission intensity to attribute most fevers to malaria. As the symptoms of malaria and acute respiratory infections overlap, VA diagnoses may simply not be accurate enough [13,14,38-41]. Moreover, this finding may be the result of a higher proportion of deaths among young children being attributable to malaria in areas of high transmission intensity as well as the lower specificity of verbal autopsy diagnosis for malaria with increasing proportional malaria mortality and the accompanying lower sensitivity for other causes of deaths in such settings [11,14,42]. The validity of the malaria diagnoses in this study is to a certain degree supported by the peak all-cause and malaria mortality occurring in the second half of infancy, the time young children are known to be at highest risk for severe malaria and deaths in hyper/holoendemic areas but not in mesoendemic areas [19,43,44]. Moreover, most deaths and also most malaria deaths were reported during the rainy season and from the holoendemic rural CRSN study area. As Nouna town is showing a mesoendemic transmission pattern and as the incidence of falciparum malaria is estimated to be lower in mesoendemic compared to holoendemic areas, this supports again the validity of the current findings [42]. The proportion of deaths attributed to diseases other than malaria is surprisingly low in this study. Beside being at least partly due to the above mentioned misclassification bias, this is also explained by the high vaccination coverage in Burkina Faso (DTP3 and measles coverage of 70% in 2003) [45] an effect also shown in other studies [17-19,46].

The VA diagnoses could not be tested against "gold standard" hospital data, mainly because very few patients are seen at the Nouna hospital, particularly during the rainy season [9,13,14,21,47]. Nevertheless, the VA questionnaire used here is consistent in itself and one can assume that the quality of coding was constant since mortality rates do not substantially vary over the years, and inter-observer agreement is good (κ = 0.74).

The inclusion of people from Nouna town and two villages in the DSS area in the year 2000 greatly improved the information content of the DSS database in terms of possible comparisons between rural and urban settings, and person-years of observation. As urbanization is increasing rapidly also in sub-Saharan Africa, more data on urban-rural comparisons are needed for the future. Therefore, it was decided to include them in this analysis although the time interval is shorter than that of the previously included 39 villages.

In conclusion, the results from this study confirm the high impact of malaria on mortality in young children living in malaria high transmission areas of SSA, and call for reinforcement the existing effective malaria control tools such as insecticide-treated bed nets and artemisinin-based combination therapy.

## Competing interests

The author(s) declare that they have no competing interests.

## Authors' contributions

The present study was designed by GPH, FS and HB. BK conceived and implemented the DSS that is source of the data used here, and contributed to the discussion. FS, GPH and OM developed the categorization of causes of death, and devised the best way to present the data. GPH and GWKanalysed the data. GWK, GPH and OM drafted the manuscript. All authors contributed to writing the paper. All authors read and approved the final manuscript.
